# 2-Ethyl-6,6-ethyl­enedisulfanediyl-7-methoxy­methyl-1,2,3,4,5,6-hexa­hydro-1,5-methano­azocino[4,3-*b*]indol-3-one

**DOI:** 10.1107/S160053681000067X

**Published:** 2010-01-09

**Authors:** Barış Tercan, Ertan Şahin, Süleyman Patır, Tuncer Hökelek

**Affiliations:** a Karabük University, Department of Physics, 78050 Karabük, Turkey; b Atatürk University, Department of Chemistry, 22240 Erzurum, Turkey; c Hacettepe University, Department of Chemistry Education, Faculty of Education, 06800 Beytepe, Ankara, Turkey; d Hacettepe University, Department of Physics, 06800 Beytepe, Ankara, Turkey

## Abstract

The title compound, C_20_H_24_N_2_O_2_S_2_, consists of a tetra­cyclic ring system containing an azocino skeleton with ethyl, dithiol­ane and methoxy­methyl groups as substituents. The benzene and five-membered rings are nearly coplanar, with a dihedral angle of 2.78 (11)°. The dithiol­ane ring adopts an envelope conformation. In the crystal structure, inter­molecular C—H⋯O hydrogen bonds link the mol­ecules into chains nearly parallel to the *c* axis. Two C—H⋯π inter­actions are also present.

## Related literature

For considerations of the hexahydro-1,5-methanoazo­cino[4,3-*b*]indole core structure as a synthetic precursor for most of the pentacyclic and tetracyclic indole alkaloids of biological interest, see: Hesse (2002 ▶); Bosch & Bonjoch (1988 ▶); Saxton (1983 ▶). For related structures, see: Hökelek *et al.* (2004 ▶, 2006 ▶, 2007 ▶); Uludağ *et al.* (2006 ▶).
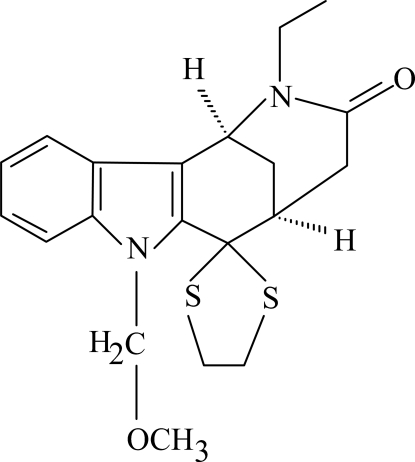


## Experimental

### 

#### Crystal data

C_20_H_24_N_2_O_2_S_2_
*M* *_r_* = 388.53
Monoclinic, 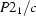

*a* = 14.0409 (3) Å
*b* = 6.8916 (2) Å
*c* = 20.2820 (4) Å
β = 109.783 (2)°
*V* = 1846.74 (8) Å^3^
*Z* = 4
Mo *K*α radiation
μ = 0.31 mm^−1^
*T* = 294 K
0.35 × 0.25 × 0.20 mm

#### Data collection

Rigaku R-AXIS RAPID-S diffractometer
Absorption correction: multi-scan (Blessing, 1995 ▶) *T*_min_ = 0.910, *T*_max_ = 0.941
24759 measured reflections
3794 independent reflections
2746 reflections with *I* > 2σ(*I*)
*R*_int_ = 0.083

#### Refinement

*R*[*F* ^2^ > 2σ(*F* ^2^)] = 0.057
*wR*(*F* ^2^) = 0.161
*S* = 1.05
3794 reflections
237 parameters
H-atom parameters constrained
Δρ_max_ = 0.61 e Å^−3^
Δρ_min_ = −0.33 e Å^−3^

### 

Data collection: *CrystalClear* (Rigaku/MSC, 2005 ▶); cell refinement: *CrystalClear*; data reduction: *CrystalClear*; program(s) used to solve structure: *SHELXS97* (Sheldrick, 2008 ▶); program(s) used to refine structure: *SHELXL97* (Sheldrick, 2008 ▶); molecular graphics: *ORTEP-3 for Windows* (Farrugia, 1997 ▶); software used to prepare material for publication: *WinGX* (Farrugia, 1999 ▶).

## Supplementary Material

Crystal structure: contains datablocks I, global. DOI: 10.1107/S160053681000067X/xu2717sup1.cif

Structure factors: contains datablocks I. DOI: 10.1107/S160053681000067X/xu2717Isup2.hkl

Additional supplementary materials:  crystallographic information; 3D view; checkCIF report

## Figures and Tables

**Table 1:** Hydrogen-bond geometry (Å, °)

*D*—H⋯*A*	*D*—H	H⋯*A*	*D*⋯*A*	*D*—H⋯*A*
C18—H18*C*⋯O1^i^	0.96	2.43	3.365 (5)	165
C11—H11⋯*Cg*1^ii^	0.93	2.80	3.569 (4)	141
C16—H16*A*⋯*Cg*1^iii^	0.96	2.66	3.514 (5)	148

Symmetry codes: (i) 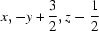; (ii) 
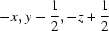
; (iii) 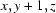. *Cg*1 is the centroid of the C7*A*/C8–C11/C11*A* ring.

**Fig. 1.**
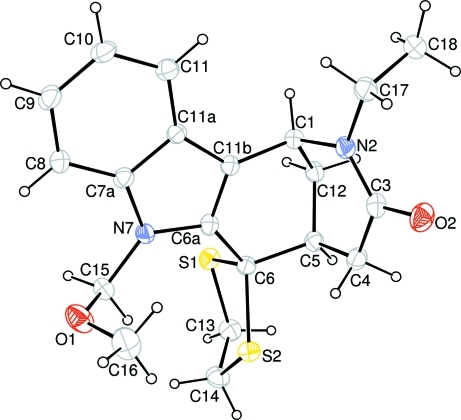
The molecular structure of the title molecule with the atom-numbering scheme. Displacement ellipsoids are drawn at the 50% probability level

**Fig. 2.**
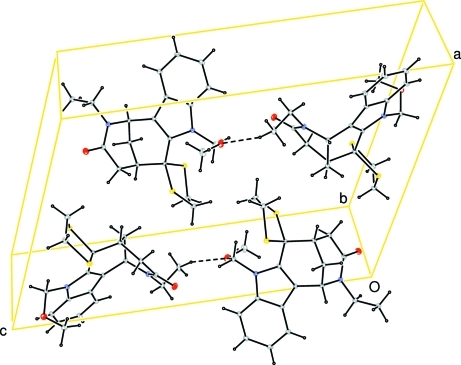
A partial packing diagram of the title compound. Hydrogen bonds are shown as dashed lines.

## supplementary crystallographic information

### Comment

The hexahydro-1,5-methano-azocino[4,3-*b*]indole core structure can be considered to be synthetic precursor for most of the pentacyclic and tetracyclic indole alkaloids of biological interests (Hesse, 2002; Bosch & Bonjoch, 1988; Saxton, 1983), such as akuminicine and uleine. Most of them have the pentacyclic ring system as a common element and include a large group of naturally occurring compounds such as strychnine, a consulvant poison.

The structures of tricyclic, tetracyclic and pentacyclic ring systems with different substituents of azocino[4,3-*b*]indole core have been determined, previously. These include *N*-(2-benzyloxyethyl)-4,7-dimethyl-6-(1,3-dithiolan- 2yl)-1,2,3,4,5,6-hexahydro-1,5-methano-2-azocino[4,3-*b*]indole-2-one, (II) (Hökelek *et al.*, 2004), 12-ethyl-2-methyl-6,6-ethylenedithio-1,2,3,4,5,6 -hexahydro-1,5-methano-2-azocino[4,3-*b*]indole-3-one, (III) (Uludağ *et al.*, 2006), 4-ethyl-6,6-ethylenedithio-2-(2-methoxymethyl)-7-methoxymethylene-2, 3,4,5,6,7-hexahydro-1,5-methano-1*H*-azocino[4,3-*b*]indole-3-one, (IV) (Hökelek *et al.*, 2006) and 2-(2,2-dimethoxyethyl)-3-oxo-1,2,3,4,5,6 -hexahydro-1,5-methano-7*H*-azocino[4,3-*b*]indole, (V) (Hökelek *et al.*, 2007). The present study was undertaken to ascertain the crystal structure of the title compound, (I).

The molecule of the title compound, (I), (Fig. 1) consists of a tetracyclic system containing an azocino skeleton with ethyl, dithiolane and methoxy methylene groups as substituents at positions N2, 6 and N7, respectively. The bonds N7—C6a [1.398 (3) Å] and N7—C7a [1.387 (3) Å] agree well with those in compounds (II) [1.392 (8) and 1.370 (8) Å], (IV) [1.393 (4) and 1.386 (5) Å] and (V) [1.377 (3) and 1.376 (3) Å]. In all four structures atom N7 is substituted. The absolute configurations of C1 and C5 are *S* and *S* (Fig. 1). The S atoms of the dithiolane ring have electron-releasing properties, but the N atom at position 7 and the O atom attached to C3 have electron-withdrawing properties, leading to some changes in the bond lengths and angles of the carbazole skeleton.

An examination of the deviations from the least-squares planes through individual rings shows that rings A (C7a/C8/C9/C10/C11/C11*a*) and B (N7/C7a/C11*a*/C11*b*/C6a) are planar. They are also coplanar with a dihedral angle of A/B = 2.78 (11)°. Rings C (C1/C11*b*/C6a/C6/C5/C12), D (C1/N2/C3/C4/C5/C12) and E (C6/S1/S2/C13/C14) are, of course, not planar. Atom C12 deviates from the planes of F(C1/C5/C6/C6a/C11*b*) and G (C1/N2/C3/C4/C5) by -0.718 (3) Å and 0.747 (3) Å, respectively where the dihedral angle between planes of F and G is F/G = 68.92 (10)°. On the other hand, the dihedral angles between the plane of H (C1/C5/C12) and the planes of F and G are 54.95 (20)° and 56.61 (20)°, respectively. Ring E has a local pseudo-mirror plane running through C13 and the midpoint of the C6—S2 bond. The conformation of ring E is an envelope, with atom C13 at the flap position, 0.729 (3) Å from the mean plane through the other four atoms.

In the crystal structure, intermolecular C—H···O hydrogen bonds (Table 1) link the molecules into chains nearly parallel to *c* axis (Fig. 2), in which they may be effective in the stabilization of the structure. There are also two C—H···π interactions (Table 1).

### Experimental

The title compound, (I), was prepared from sodium hydride (48.0 mg, 2.00 mmol) and 6-(1,3-dithiolan-2-yl)-1,2,3,4,5,6-hexahydro-1,5-methano-azocino[4,3–6] indole-3-one (500.0 mg, 1.38 mmol) in THF (40 ml) and bromoethane (5 ml). The mixture was heated at reflux for 4 h under nitrogen atmosphere. Later the mixture was cooled in an ice bath and methanol (5 ml) and water (25 ml) were added. After extraction with ethyl acetate (30 ml), the organic layer was dried with Na_2_SO_4_ and the solvent was evaporated. The residue was crystallized from aceton (yield; 450.0 mg, 83%), m.p. 469 K.

### Crystal data

**Table:**

C_20_H_24_N_2_O_2_S_2_	*F*(000) = 824
*M**_r_* = 388.53	*D*_x_ = 1.397 Mg m^−^^3^
Monoclinic, *P*2_1_/*c*	Mo *K*α radiation, λ = 0.71073 Å
Hall symbol: -P 2ybc	Cell parameters from 6800 reflections
*a* = 14.0409 (3) Å	θ = 2.1–26.4°
*b* = 6.8916 (2) Å	µ = 0.31 mm^−^^1^
*c* = 20.2820 (4) Å	*T* = 294 K
β = 109.783 (2)°	Block, colorless
*V* = 1846.74 (8) Å^3^	0.35 × 0.25 × 0.20 mm
*Z* = 4	

### Data collection

**Table:**

Rigaku R-AXIS RAPID-S diffractometer	3794 independent reflections
Radiation source: fine-focus sealed tube	2746 reflections with *I* > 2σ(*I*)
graphite	*R*_int_ = 0.083
ω scans	θ_max_ = 26.4°, θ_min_ = 2.2°
Absorption correction: multi-scan (Blessing, 1995)	*h* = −17→17
*T*_min_ = 0.910, *T*_max_ = 0.941	*k* = −8→7
24759 measured reflections	*l* = −25→25

### Refinement

**Table:**

Refinement on *F*^2^	Primary atom site location: structure-invariant direct methods
Least-squares matrix: full	Secondary atom site location: difference Fourier map
*R*[*F*^2^ > 2σ(*F*^2^)] = 0.057	Hydrogen site location: inferred from neighbouring sites
*wR*(*F*^2^) = 0.161	H-atom parameters constrained
*S* = 1.05	*w* = 1/[σ^2^(*F*_o_^2^) + (0.0667*P*)^2^ + 0.9643*P*] where *P* = (*F*_o_^2^ + 2*F*_c_^2^)/3
3794 reflections	(Δ/σ)_max_ < 0.001
237 parameters	Δρ_max_ = 0.61 e Å^−^^3^
0 restraints	Δρ_min_ = −0.33 e Å^−^^3^

### Special details

**Table:**

Geometry. All e.s.d.'s (except the e.s.d. in the dihedral angle between two l.s. planes) are estimated using the full covariance matrix. The cell e.s.d.'s are taken into account individually in the estimation of e.s.d.'s in distances, angles and torsion angles; correlations between e.s.d.'s in cell parameters are only used when they are defined by crystal symmetry. An approximate (isotropic) treatment of cell e.s.d.'s is used for estimating e.s.d.'s involving l.s. planes.
Refinement. Refinement of *F*^2^ against ALL reflections. The weighted *R*-factor *wR* and goodness of fit *S* are based on *F*^2^, conventional *R*-factors *R* are based on *F*, with *F* set to zero for negative *F*^2^. The threshold expression of *F*^2^ > σ(*F*^2^) is used only for calculating *R*-factors(gt) *etc*. and is not relevant to the choice of reflections for refinement. *R*-factors based on *F*^2^ are statistically about twice as large as those based on *F*, and *R*- factors based on ALL data will be even larger.

### Fractional atomic coordinates and isotropic or equivalent isotropic displacement parameters (Å^2^)

**Table:**

	*x*	*y*	*z*	*U*_iso_*/*U*_eq_	
S1	0.47776 (6)	0.49118 (12)	0.40721 (4)	0.0511 (2)	
S2	0.44792 (6)	0.89818 (12)	0.35693 (4)	0.0509 (2)	
O1	0.2345 (2)	0.9462 (4)	0.44738 (13)	0.0768 (8)	
O2	0.23070 (18)	0.8019 (4)	0.09759 (11)	0.0689 (7)	
C1	0.2432 (2)	0.4188 (4)	0.22142 (14)	0.0436 (7)	
H1	0.2104	0.2915	0.2114	0.052*	
N2	0.20617 (18)	0.5406 (4)	0.15768 (11)	0.0473 (6)	
C3	0.2592 (2)	0.6945 (5)	0.14953 (15)	0.0515 (8)	
C4	0.3593 (2)	0.7400 (5)	0.20602 (15)	0.0538 (8)	
H4A	0.3509	0.8600	0.2284	0.065*	
H4B	0.4084	0.7649	0.1831	0.065*	
C5	0.4061 (2)	0.5879 (4)	0.26479 (14)	0.0434 (7)	
H5	0.4782	0.5755	0.2709	0.052*	
C6	0.3979 (2)	0.6464 (4)	0.33652 (13)	0.0407 (6)	
C6A	0.2908 (2)	0.6149 (4)	0.33343 (14)	0.0418 (6)	
N7	0.25017 (17)	0.6624 (4)	0.38550 (11)	0.0436 (6)	
C7A	0.1539 (2)	0.5828 (4)	0.36673 (15)	0.0442 (7)	
C8	0.0862 (2)	0.5820 (5)	0.40350 (17)	0.0528 (8)	
H8	0.1008	0.6456	0.4462	0.063*	
C9	−0.0033 (3)	0.4828 (5)	0.37369 (19)	0.0591 (9)	
H9	−0.0499	0.4783	0.3971	0.071*	
C10	−0.0259 (2)	0.3890 (5)	0.30934 (18)	0.0576 (8)	
H10	−0.0876	0.3251	0.2905	0.069*	
C11	0.0409 (2)	0.3887 (5)	0.27301 (16)	0.0509 (7)	
H11	0.0251	0.3257	0.2301	0.061*	
C11A	0.1336 (2)	0.4859 (4)	0.30238 (14)	0.0413 (6)	
C11B	0.2214 (2)	0.5078 (4)	0.28250 (14)	0.0401 (6)	
C12	0.3567 (2)	0.3922 (4)	0.24135 (15)	0.0469 (7)	
H12A	0.3732	0.3452	0.2015	0.056*	
H12B	0.3807	0.2984	0.2790	0.056*	
C13	0.5895 (2)	0.6397 (5)	0.42991 (17)	0.0583 (8)	
H13A	0.6373	0.6000	0.4748	0.070*	
H13B	0.6218	0.6271	0.3948	0.070*	
C14	0.5567 (2)	0.8471 (6)	0.43355 (17)	0.0620 (9)	
H14A	0.6116	0.9348	0.4353	0.074*	
H14B	0.5396	0.8660	0.4756	0.074*	
C15	0.2919 (2)	0.7819 (5)	0.44770 (15)	0.0497 (7)	
H15A	0.2968	0.7047	0.4887	0.060*	
H15B	0.3598	0.8216	0.4514	0.060*	
C16	0.2197 (4)	1.0751 (6)	0.3929 (2)	0.0853 (13)	
H16A	0.1754	1.1772	0.3968	0.128*	
H16B	0.1898	1.0086	0.3492	0.128*	
H16C	0.2835	1.1293	0.3948	0.128*	
C17	0.1124 (2)	0.4840 (5)	0.10217 (16)	0.0575 (9)	
H17A	0.0824	0.5974	0.0746	0.069*	
H17B	0.0647	0.4343	0.1232	0.069*	
C18	0.1315 (3)	0.3308 (6)	0.05469 (17)	0.0741 (11)	
H18A	0.0682	0.2879	0.0219	0.111*	
H18B	0.1661	0.2227	0.0824	0.111*	
H18C	0.1725	0.3846	0.0297	0.111*	

### Atomic displacement parameters (Å^2^)

**Table:**

	*U*^11^	*U*^22^	*U*^33^	*U*^12^	*U*^13^	*U*^23^
S1	0.0460 (4)	0.0572 (5)	0.0457 (4)	0.0018 (3)	0.0099 (3)	0.0075 (3)
S2	0.0487 (4)	0.0488 (5)	0.0541 (5)	−0.0058 (3)	0.0160 (3)	−0.0024 (3)
O1	0.100 (2)	0.0602 (17)	0.0658 (16)	0.0158 (15)	0.0232 (14)	−0.0083 (12)
O2	0.0691 (15)	0.0801 (19)	0.0538 (13)	−0.0012 (13)	0.0160 (11)	0.0208 (12)
C1	0.0472 (16)	0.0431 (17)	0.0419 (15)	−0.0030 (13)	0.0168 (12)	−0.0012 (12)
N2	0.0470 (13)	0.0569 (17)	0.0367 (12)	−0.0035 (12)	0.0125 (10)	−0.0003 (11)
C3	0.0524 (17)	0.063 (2)	0.0435 (16)	0.0038 (15)	0.0217 (13)	0.0023 (14)
C4	0.0538 (17)	0.061 (2)	0.0460 (16)	−0.0086 (15)	0.0165 (14)	0.0063 (14)
C5	0.0377 (14)	0.0506 (18)	0.0442 (15)	0.0010 (12)	0.0168 (12)	−0.0017 (12)
C6	0.0365 (14)	0.0443 (17)	0.0391 (14)	−0.0008 (12)	0.0098 (11)	0.0002 (12)
C6A	0.0386 (14)	0.0500 (18)	0.0384 (14)	0.0034 (12)	0.0149 (11)	0.0026 (12)
N7	0.0419 (13)	0.0487 (15)	0.0428 (12)	−0.0032 (11)	0.0178 (10)	−0.0072 (10)
C7A	0.0425 (15)	0.0429 (17)	0.0499 (16)	−0.0001 (12)	0.0192 (13)	0.0001 (12)
C8	0.0562 (18)	0.055 (2)	0.0564 (18)	−0.0026 (15)	0.0308 (15)	−0.0066 (14)
C9	0.0571 (19)	0.056 (2)	0.077 (2)	−0.0016 (16)	0.0402 (17)	−0.0018 (16)
C10	0.0460 (17)	0.056 (2)	0.074 (2)	−0.0104 (15)	0.0246 (16)	−0.0030 (16)
C11	0.0472 (16)	0.0501 (19)	0.0553 (18)	−0.0036 (14)	0.0174 (14)	−0.0013 (14)
C11A	0.0418 (14)	0.0400 (16)	0.0442 (15)	−0.0020 (12)	0.0171 (12)	−0.0002 (12)
C11B	0.0390 (14)	0.0433 (17)	0.0391 (14)	0.0010 (12)	0.0147 (11)	0.0007 (11)
C12	0.0475 (16)	0.052 (2)	0.0429 (15)	0.0015 (14)	0.0180 (13)	−0.0029 (13)
C13	0.0432 (16)	0.068 (2)	0.0566 (19)	0.0007 (15)	0.0073 (14)	−0.0007 (16)
C14	0.0479 (17)	0.074 (3)	0.0577 (19)	−0.0118 (16)	0.0099 (15)	−0.0141 (17)
C15	0.0520 (17)	0.0524 (19)	0.0441 (16)	0.0017 (14)	0.0156 (13)	−0.0071 (13)
C16	0.119 (4)	0.063 (3)	0.074 (3)	0.027 (2)	0.035 (2)	0.013 (2)
C17	0.0477 (17)	0.074 (2)	0.0442 (17)	−0.0052 (16)	0.0076 (13)	0.0027 (15)
C18	0.086 (3)	0.086 (3)	0.0510 (19)	−0.031 (2)	0.0245 (18)	−0.0132 (19)

### Geometric parameters (Å, °)

**Table:**

S1—C6	1.835 (3)	C9—H9	0.9300
S1—C13	1.798 (3)	C10—C9	1.393 (5)
S2—C6	1.866 (3)	C10—H10	0.9300
S2—C14	1.807 (3)	C11—C10	1.375 (4)
O1—C15	1.388 (4)	C11—H11	0.9300
O1—C16	1.377 (4)	C11A—C11	1.405 (4)
O2—C3	1.238 (4)	C11B—C1	1.504 (4)
C1—C12	1.516 (4)	C11B—C11A	1.429 (4)
C1—H1	0.9800	C12—C5	1.518 (4)
N2—C1	1.480 (4)	C12—H12A	0.9700
N2—C3	1.338 (4)	C12—H12B	0.9700
N2—C17	1.466 (4)	C13—H13A	0.9700
C3—C4	1.515 (4)	C13—H13B	0.9700
C4—H4A	0.9700	C14—C13	1.511 (5)
C4—H4B	0.9700	C14—H14A	0.9700
C5—C4	1.556 (4)	C14—H14B	0.9700
C5—H5	0.9800	C15—H15A	0.9700
C6—C5	1.551 (4)	C15—H15B	0.9700
C6A—C6	1.500 (4)	C16—H16A	0.9600
C6A—C11B	1.371 (4)	C16—H16B	0.9600
N7—C6A	1.398 (3)	C16—H16C	0.9600
N7—C7A	1.387 (3)	C17—C18	1.513 (5)
N7—C15	1.454 (4)	C17—H17A	0.9700
C7A—C8	1.392 (4)	C17—H17B	0.9700
C7A—C11A	1.407 (4)	C18—H18A	0.9600
C8—C9	1.378 (5)	C18—H18B	0.9600
C8—H8	0.9300	C18—H18C	0.9600
			
C13—S1—C6	96.77 (14)	C10—C11—C11A	118.3 (3)
C14—S2—C6	98.91 (15)	C10—C11—H11	120.8
C16—O1—C15	117.4 (3)	C11A—C11—H11	120.8
N2—C1—C11B	112.3 (2)	C7A—C11A—C11B	106.8 (2)
N2—C1—C12	109.2 (2)	C11—C11A—C7A	119.2 (3)
N2—C1—H1	108.9	C11—C11A—C11B	134.0 (3)
C11B—C1—C12	108.6 (2)	C6A—C11B—C1	123.3 (2)
C11B—C1—H1	108.9	C6A—C11B—C11A	107.8 (2)
C12—C1—H1	108.9	C11A—C11B—C1	128.7 (2)
C3—N2—C1	120.8 (2)	C1—C12—C5	107.7 (2)
C3—N2—C17	120.6 (3)	C1—C12—H12A	110.2
C17—N2—C1	118.6 (3)	C1—C12—H12B	110.2
O2—C3—N2	123.1 (3)	C5—C12—H12A	110.2
O2—C3—C4	118.0 (3)	C5—C12—H12B	110.2
N2—C3—C4	118.9 (3)	H12A—C12—H12B	108.5
C3—C4—C5	118.9 (3)	S1—C13—H13A	110.3
C3—C4—H4A	107.6	S1—C13—H13B	110.3
C3—C4—H4B	107.6	C14—C13—S1	107.3 (2)
C5—C4—H4A	107.6	C14—C13—H13A	110.3
C5—C4—H4B	107.6	C14—C13—H13B	110.3
H4A—C4—H4B	107.0	H13A—C13—H13B	108.5
C4—C5—H5	107.8	S2—C14—H14A	109.9
C6—C5—C4	113.4 (2)	S2—C14—H14B	109.9
C6—C5—H5	107.8	C13—C14—S2	109.0 (2)
C12—C5—C4	109.2 (2)	C13—C14—H14A	109.9
C12—C5—C6	110.7 (2)	C13—C14—H14B	109.9
C12—C5—H5	107.8	H14A—C14—H14B	108.3
S1—C6—S2	106.50 (13)	O1—C15—N7	113.3 (2)
C5—C6—S1	111.18 (19)	O1—C15—H15A	108.9
C5—C6—S2	107.92 (19)	O1—C15—H15B	108.9
C6A—C6—S1	106.70 (19)	N7—C15—H15A	108.9
C6A—C6—S2	116.2 (2)	N7—C15—H15B	108.9
C6A—C6—C5	108.4 (2)	H15A—C15—H15B	107.7
N7—C6A—C6	126.4 (2)	O1—C16—H16A	109.5
C11B—C6A—N7	109.2 (2)	O1—C16—H16B	109.5
C11B—C6A—C6	123.8 (2)	O1—C16—H16C	109.5
C6A—N7—C15	129.9 (2)	H16A—C16—H16B	109.5
C7A—N7—C6A	108.0 (2)	H16A—C16—H16C	109.5
C7A—N7—C15	122.1 (2)	H16B—C16—H16C	109.5
N7—C7A—C8	129.2 (3)	N2—C17—C18	111.5 (3)
N7—C7A—C11A	108.4 (2)	N2—C17—H17A	109.3
C8—C7A—C11A	122.3 (3)	N2—C17—H17B	109.3
C7A—C8—H8	121.5	C18—C17—H17A	109.3
C9—C8—C7A	117.0 (3)	C18—C17—H17B	109.3
C9—C8—H8	121.5	H17A—C17—H17B	108.0
C8—C9—C10	121.7 (3)	C17—C18—H18A	109.5
C8—C9—H9	119.1	C17—C18—H18B	109.5
C10—C9—H9	119.1	C17—C18—H18C	109.5
C9—C10—H10	119.3	H18A—C18—H18B	109.5
C11—C10—C9	121.5 (3)	H18A—C18—H18C	109.5
C11—C10—H10	119.3	H18B—C18—H18C	109.5
			
C13—S1—C6—S2	−26.72 (17)	N7—C6A—C11B—C1	−175.0 (3)
C13—S1—C6—C5	90.6 (2)	N7—C6A—C11B—C11A	0.0 (3)
C13—S1—C6—C6A	−151.4 (2)	C6—C6A—C11B—C1	−3.6 (4)
C6—S1—C13—C14	45.6 (2)	C6—C6A—C11B—C11A	171.4 (3)
C14—S2—C6—S1	4.39 (17)	C7A—N7—C6A—C6	−170.9 (3)
C14—S2—C6—C5	−115.1 (2)	C7A—N7—C6A—C11B	0.2 (3)
C14—S2—C6—C6A	123.0 (2)	C15—N7—C6A—C6	12.6 (5)
C6—S2—C14—C13	25.5 (3)	C15—N7—C6A—C11B	−176.2 (3)
C16—O1—C15—N7	−59.0 (4)	C6A—N7—C7A—C8	176.2 (3)
N2—C1—C12—C5	67.7 (3)	C6A—N7—C7A—C11A	−0.4 (3)
C11B—C1—C12—C5	−55.0 (3)	C15—N7—C7A—C8	−7.0 (5)
C3—N2—C1—C11B	80.0 (3)	C15—N7—C7A—C11A	176.4 (3)
C3—N2—C1—C12	−40.5 (4)	C6A—N7—C15—O1	116.8 (3)
C17—N2—C1—C11B	−102.7 (3)	C7A—N7—C15—O1	−59.2 (4)
C17—N2—C1—C12	136.8 (3)	N7—C7A—C8—C9	−176.8 (3)
C1—N2—C3—O2	−179.1 (3)	C11A—C7A—C8—C9	−0.6 (5)
C1—N2—C3—C4	1.3 (4)	N7—C7A—C11A—C11	178.5 (3)
C17—N2—C3—O2	3.6 (5)	N7—C7A—C11A—C11B	0.4 (3)
C17—N2—C3—C4	−175.9 (3)	C8—C7A—C11A—C11	1.6 (5)
C1—N2—C17—C18	−82.9 (3)	C8—C7A—C11A—C11B	−176.5 (3)
C3—N2—C17—C18	94.4 (4)	C7A—C8—C9—C10	−0.7 (5)
O2—C3—C4—C5	−169.0 (3)	C11—C10—C9—C8	1.0 (6)
N2—C3—C4—C5	10.6 (4)	C11A—C11—C10—C9	0.0 (5)
C6—C5—C4—C3	−106.4 (3)	C7A—C11A—C11—C10	−1.3 (5)
C12—C5—C4—C3	17.5 (4)	C11B—C11A—C11—C10	176.2 (3)
S1—C6—C5—C4	−167.51 (19)	C6A—C11B—C1—N2	−97.1 (3)
S1—C6—C5—C12	69.4 (3)	C6A—C11B—C1—C12	23.8 (4)
S2—C6—C5—C4	−51.1 (3)	C11A—C11B—C1—N2	89.0 (4)
S2—C6—C5—C12	−174.13 (19)	C11A—C11B—C1—C12	−150.2 (3)
C6A—C6—C5—C4	75.5 (3)	C1—C11B—C11A—C7A	174.4 (3)
C6A—C6—C5—C12	−47.5 (3)	C1—C11B—C11A—C11	−3.3 (5)
N7—C6A—C6—S1	64.9 (3)	C6A—C11B—C11A—C7A	−0.3 (3)
N7—C6A—C6—S2	−53.6 (4)	C6A—C11B—C11A—C11	−177.9 (3)
N7—C6A—C6—C5	−175.3 (3)	C1—C12—C5—C4	−54.7 (3)
C11B—C6A—C6—S1	−105.0 (3)	C1—C12—C5—C6	70.7 (3)
C11B—C6A—C6—S2	136.5 (3)	S2—C14—C13—S1	−46.9 (3)
C11B—C6A—C6—C5	14.8 (4)		

### Hydrogen-bond geometry (Å, °)

**Table:**

*D*—H···*A*	*D*—H	H···*A*	*D*···*A*	*D*—H···*A*
C18—H18C···O1^i^	0.96	2.43	3.365 (5)	165
C11—H11···Cg1^ii^	0.93	2.80	3.569 (4)	141
C16—H16A···Cg1^iii^	0.96	2.66	3.514 (5)	148

Symmetry codes: (i) *x*, −*y*+3/2, *z*−1/2; (ii) −*x*, *y*−1/2, −*z*+1/2; (iii) *x*, *y*+1, *z*.
